# RNA-Seq transcriptome analysis of Spirodela dormancy without reproduction

**DOI:** 10.1186/1471-2164-15-60

**Published:** 2014-01-23

**Authors:** Wenqin Wang, Yongrui Wu, Joachim Messing

**Affiliations:** 1 Waksman Institute of Microbiology, Rutgers, The State University of New Jersey, 190 Frelinghuysen Road, Piscataway, NJ, USA

**Keywords:** Dormancy, ABA signaling, Oil crop, RNA-Seq, Next-generation sequencing, Whole-genome gene expression analysis

## Abstract

**Background:**

Higher plants exhibit a remarkable phenotypic plasticity to adapt to adverse environmental changes. The Greater Duckweed Spirodela, as an aquatic plant, presents exceptional tolerance to cold winters through its dormant structure of turions in place of seeds. Abundant starch in turions permits them to sink and escape the freezing surface of waters. Due to their clonal propagation, they are the fastest growing biomass on earth, providing yet an untapped source for industrial applications.

**Results:**

We used next generation sequencing technology to examine the transcriptome of turion development triggered by exogenous ABA. A total of 208 genes showed more than a 4-fold increase compared with 154 down-regulated genes in developing turions. The analysis of up-regulated differential expressed genes in response to dormancy exposed an enriched interplay among various pathways: signal transduction, seed dehydration, carbohydrate and secondary metabolism, and senescence. On the other side, the genes responsible for rapid growth and biomass accumulation through DNA assembly, protein synthesis and carbon fixation are repressed. Noticeably, three members of late embryogenesis abundant protein family are exclusively expressed during turion formation. High expression level of key genes in starch synthesis are APS1, APL3 and GBSSI, which could artificially be reduced for re-directing carbon flow from photosynthesis to create a higher energy biomass.

**Conclusions:**

The identification and functional annotation of differentially expressed genes open a major step towards understanding the molecular network underlying vegetative frond dormancy. Moreover, genes have been identified that could be engineered in duckweeds for practical applications easing agricultural production of food crops.

## Background

Plants, unlike animals, do not have a fur or can seek shelter to survive under food shortage and cold weather. Consequently, they adapt to dormancy to avoid adverse environments, such as poor nutrition, chilling temperature, and drought. Dormancy is a complex state of plant development, in which the plant body exhibits little or no growth. They recover their growth once the conditions are favorable.

There are mainly two types of plant dormancy by forming seeds or buds. Seed dormancy has been observed for many plants species including our major crops [1-3]. Winter dormant buds are found for instance in woody plants, bulbs, rhizomes and tubers of herbaceous plants [4]. Studies on the molecular mechanisms of bud dormancy transitions in perennial woody plants have been conducted, including pear [5], oak [6], and poplar [7].

*Spirodela polyrhiza*, a floating aquatic monocot, develops a specific dormant organ called turion during its life cycle, which alternates between periods of clonal propagation and dormancy. Its leaf, stem and bud are extremely compact in form of a round-shaped frond, resembling a single leaf. Large numbers of Spirodela plants can be maintained like cell cultures under totally controlled medium and environmental conditions. They reproduce vegetatively through budding of fronds (growth phase) during spring and summer [8] and transition to turions (dormant phase), when there is shortage of nutrition in the fall or the temperature drops in the winter [9]. Noticeably, fronds perform photosynthesis and turions function as storage for starch and germinate in the following spring [10-13]. Turion cells exhibit dense intercellular space, thick cell wall and are also rich in anthocyanins [14]. Therefore, turions provide a unique system to study both bud and seed dormancy because they reproduce like buds without sexual hybridization but functionally are equivalent to seeds that could generate a progeny plant in the growing season. Previous studies have shown that addition of ABA into growth medium quickly leads to turion formation after 5 days of treatment in the laboratory [13,15,16]. Only 3 days after ABA treatment, the Spirodela primordium is irreversibly committed to turion development [15]. The ease of growth and its direct contact with water make Spirodela a model system to gain molecular insights into plant dormancy [17].

At the molecular level, some studies on turion development have already been performed. For example, the transcript level of D-myo-inositol-3-phosphate synthase is rapidly induced within 15 min of ABA application, an enzyme that plays a key role in the inositol metabolism of the cell wall [18,19]. The expression of the key enzyme ADP-glucose pyrophosphorylase (APL) for starch production [13] is significantly changed during turion formation. Still, not much information is known about the global transcriptome profiling for turion formation in this model system. To further uncover the regulation of gene expression as the phase switches, we took advantage of RNA deep sequencing, and compared the transcriptome between fronds and developing turions. A more comprehensive understanding of the gene repertoire and its regulation during turion formation has also great potential for industrial applications including the redirection of carbon flow into higher energy products.

## Methods

### Sample preparation

*Spirodela polyrhiza* 7498 was grown in half-strength Schenk and Hildebrandt basal salt mixture (Sigma, S6765) with 1% sucrose liquid medium under 16-hrs light, 8-hrs dark photoperiod. Plant tissues from four biological replicates for fronds without ABA treatment and developing turions with 3-day 10 μM ABA were collected and frozen in liquid nitrogen. 10 μg of total RNA was extracted for each sample by RNA-easy Qiagen kit with RLC buffer due to second metabolites. Ribosomal RNA was depleted with a kit from Epicenter (MRZPL116) in order to increase the coverage of other RNA classes. Vegetative fronds and turions with 14 days ABA treatment were fixed, embedded, and examined under transmission electron microscope as described [13,20].

### Library construction and sequence quality control

We started with ~300 ng rRNA-depleted total RNA, fragmented the RNA, performed reverse transcription and size-selected the cDNA, used Emulsion PCR to amplify the complex gene libraries and prevent formation of chimeric cDNA products. All steps followed the manufacturer's guide (SOLiD™ total RNA-Seq kit). To minimize potential experimental batch effect, eight samples were barcoded, pooled, and evenly distributed into three lanes. The single-end reads with the size of 75 bp were generated with our in-house SOLiD 5500 platform. The Exact Call Chemistry (ECC) module was utilized in the sequencing run, which is an optional kit that is used to further enhance sequencing accuracy by generating reference-free bases directly. After quality trimming with score of 20, reads with a minimum length of 40 bp were saved.

### Read mapping and quantifying gene expression

The remaining reads were mapped to the reference genome *Spirodela polyrhiza* 7498 (http://www.waksman.rutgers.edu/spirodela/genome; GenBank Accession #ATDW01000000), which was recently sequenced, assembled, and annotated, by using TopHat 2 [21] with Bowtie [22]. TopHat is a fast splice junction mapper for RNA-Seq reads. It aligns RNA-Seq reads to reference genomes using the ultra high-throughput short read aligner Bowtie, and then analyzes the mapping results to identify splice junctions between exons. Gene expression levels were normalized using fragments per kilobase of exon per million mapped reads (FPKM). Transcript abundance and differential gene expression were calculated with Cufflinks [23]. DE genes were defined, as when their absolute value of log2 fold change was higher than 2 and their P value was less than 0.01.

To test the validity of our measurements, we used independent data obtained in a separate study under the same induction conditions as in this study from the expression of ADP-glucose pyrophosphorylase genes with qRT-PCR [13]. We also used northern blot data of the expression of the tur4 gene obtained in yet another study [24].

### Functional annotation and *cis-*element predictions

For each DE gene, GO annotation was obtained with the program of blast2go, which uses a blast algorithm to assign GO terms to sequences based on similarity [25]. GO enrichment was performed in two groups of gene sets, respectively, one of highly expressed transcripts in turions, the other one of highly expressed transcripts in fronds based on the whole gene set of the Spirodela genome using GOseq, which adjusts the bias from gene lengths [26]. The *cis-*acting regulatory DNA elements were predicted by signal scan search from PLACE database [27]. PLACE is a database of motifs found in plant *cis*-acting regulatory DNA elements, all from previously published reports. We dissected 1-kb regions upstream of DE genes and scanned them for potential pairs of TFs and *cis*-elements.

## Results

### Calibration and selection of tissue samples

**Figure 1:**
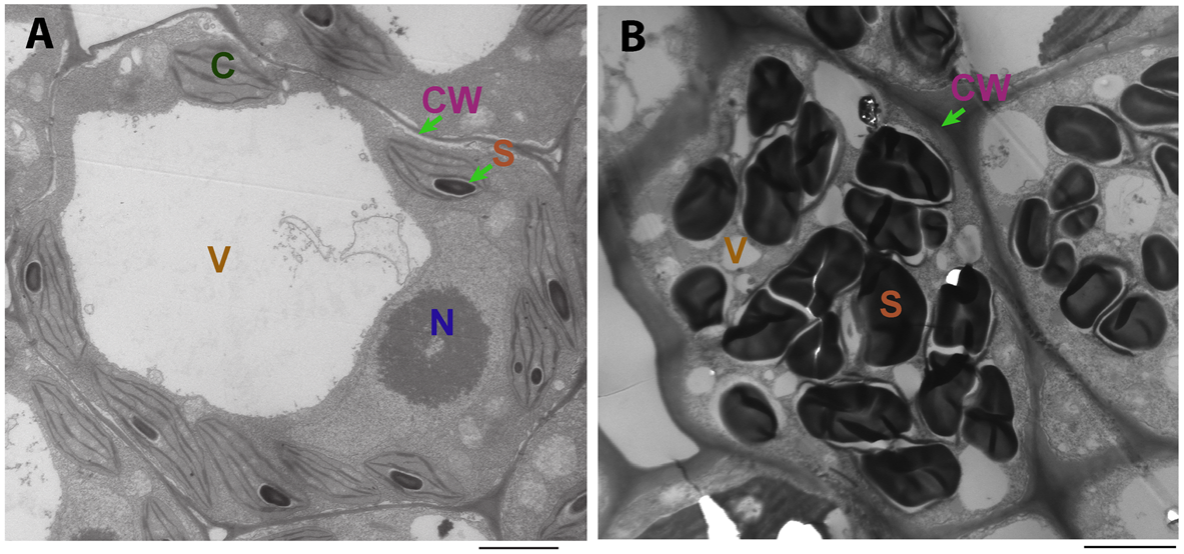
**Comparison between frond and mature turion by TEM. A**. A frond cell with a big vacuole and well-shaped chloroplasts but few and less starch granules, Bar = 2 μm; **B**. A turion cell with thick cell wall and abundant starch granules, Bar = 2 μm; Abbreviation: cell wall (CW), chloroplast (C), starch granule (S), vacuole (V) and nucleus (N).

A comprehensive study for turion formation has been done using abscisic acid (ABA) induction [14,15,17,28,29]. Three days after ABA induction, the Spirodela primordium is committed to turion development, which cannot be reversed. All primary biosynthesis of protein, mRNA and DNA are shut down resulting in the onset of the dormant state [28]. To calibrate our growing conditions with previous investigations, we used transmission electron microscopy (TEM) to investigate different developmental stages. We chose fronds and developing turions with 3 days after ABA treatment instead of 14 days because 14-day treatment is not a key transition state and RNA purification is greatly hampered by high content of starch, but mature turions with 14-days treatment provide a more complete structural image through TEM. Turion cells have thicker cell walls, multiple smaller vacuoles and distorted plastids filled with abundant starch granules, whereas frond cells differ with having well-shaped chloroplasts consistent with previous observations (Figure 1). Therefore, growing conditions and turion induction appear to be reproducible.

### Mapping RNA-Seq reads

We used eight samples in total, with each condition having four biological replicates. To eliminate potentially technical variation from biological replicates, they were multiplexed, pooled, and sequenced with the SOLiD 5500 platform. A total of 15 ~ 41 million quality reads per sample were generated after filtering raw reads (Table 1).

**Table 1:** Summary of sequence read alignments to three genome references

**Sample**	**Qualified total reads**	**Reads # map nuclear genome**	**Map nuclear genome**	**Nuclear coverage**	**Map chloroplast**	**Map mitochondria**	**Map rDNA**
Fronds 1	24,356,014	12,795,916	53%	42	35%	1%	4%
Fronds 2	41,310,111	22,039,845	53%	72	37%	3%	4%
Fronds 3	28,333,911	16,444,539	58%	54	29%	2%	6%
Fronds 4	28,188,669	16,282,775	58%	53	30%	2%	9%
Turions 1	26,484,522	15,431,023	58%	50	28%	2%	1%
Turions 2	28,466,211	16,123,639	57%	53	34%	2%	2%
Turions 3	25,754,050	15,697,393	61%	51	26%	2%	3%
Turions 4	14,996,833	8,824,987	59%	29	29%	2%	1%

The high quality reads were mapped to chloroplast [30], mitochondria [31], and nuclear genomes [32], respectively. We could clearly divide sequence reads into these three classes. Surprisingly, there was an abundance of organelle-derived transcripts with 28 ~ 39% of total reads. With this depth of data we could assemble sequences for complete plastid and mitochondrial transcriptomes. The high proportion of organelle reads stresses the important roles of their transcripts, provides us with their expression profiles and facilitates the phylogenetic analysis [33]. Based on the combined reads of nuclear and organelle RNAs, more than 89% of our RNA-Seq reads were mappable. It also suggests that part of previously unmapped reads in other studies remained undetected because of their organellar origin [5,34-36]. We still found that 1 ~ 9% of total reads were derived from ribosomal RNA, which is an indication that the protocol for the depletion of ribosomal RNA from samples was reasonably successful. Such efficiency is critical for mainly uncovering the desired transcriptome with complete coverage and in a cost-effective manner [37].

Among the total reads, 53-61% originated from nuclear DNA, lower than in other cases with about 80% of mappable sequences [34,36]. The reason could be the method we used through ribosomal RNA removal rather than polyA selection. In case of polyA selection, organelle transcripts are automatically removed due to the lack of the polyA tail in organelle transcripts, whereas most of them were captured by our method of ribosomal RNA removal. Excluding the abundant organelle and rDNA reads, nuclear reads corresponded to 29 ~ 72X coverage for all annotated genes (Table 1), demonstrating that the depth used in our study was sufficient to cover the Spirodela nuclear transcriptome.

### Identification and validation of differentially expressed genes

Comparison of frond and developing turion samples provided us with 362 differentially expressed (DE) genes. A total of 117 had greater than 10-fold difference in mRNA levels and 208 genes were up-regulated and expressed at higher levels in developing turions than in fronds, whereas 154 genes were down-regulated, indicating lower expression in turions than in fronds (Table 2).

**Table 2:** Fold change in differentially expressed genes between in fronds and developing turions at FDR < 0.01. “FC” means fold change

**Fold change**	**4≤FC≤5**	**5<FC≤10**	**10<FC≤15**	**15<FC≤20**	**FC>20**	**Sum**
Genes expressed lower in turions than fronds	37	73	12	10	22	154
Genes expressed higher in turions than fronds	38	97	25	15	33	208

**Figure 2:**
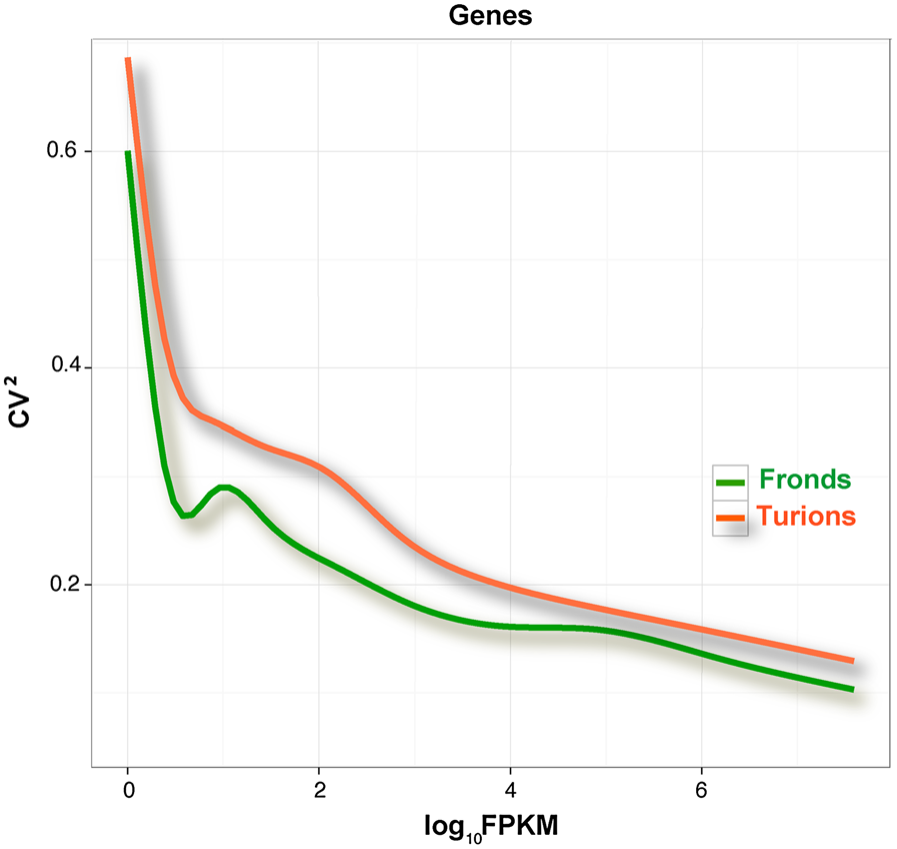
**Biological variation for biological replicates from fronds and developing turions.** Biological variation was represented by the square coefficient of variation of FPKM values for each gene (CV^2^).

Previous studies had indicated that a small number of biological replicates might not be robust enough because it is impossible to know whether expression patterns are specific to individuals or are characteristic for the total population. Even for RNA deep sequencing, a sufficient number of biological replicates are still required to have confidence in the measurements [38-40]. Because two biological replicates usually are not sufficient to account for sample variability, we increased this number to four independent biological replicates. The coefficient of variation to the power of two (CV^2^), a normalized measure of cross-replicate variability that can be useful for evaluating the quality of RNA-Seq data, was calculated to exhibit the biological variation (Figure 2). As expected, the data showed that the abundance of the genes varied between replicate RNA samples, especially for ones with lower FPKM values. In addition, four biological replicates allowed us to take variation within the target population into account and also counteract random technical variations [23,41].

**Figure 3:**
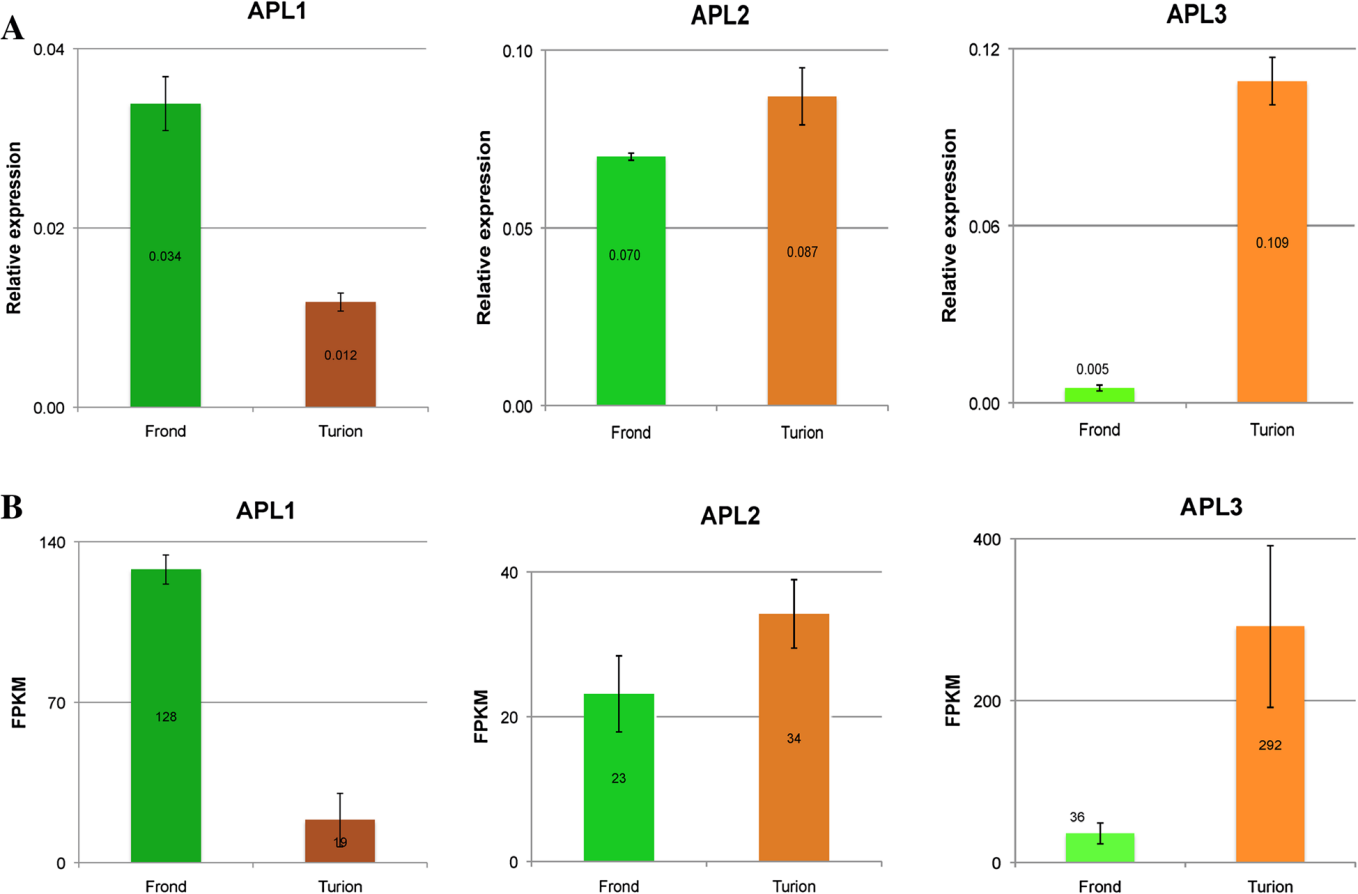
**Comparison of APL gene expression from qRT-PCR vs. RNA-Seq. A**. APL gene expression from qRT-PCR; **B**. APL gene expression from RNA-Seq data. Abbreviation: APL1-JN180634; APL2-JN180635; APL3-JN180636.

To test the validity of our measurements, we compared the RNA-Seq data of the three transcripts of ADP-glucose pyrophosphorylases (APLs) for starch synthesis with the developmental expression of these genes studied previously, which were done with qRT-PCR [13]. Indeed, the correlation co-efficient of 0.992 indicated that the two independent measurements were consistent and showed similar patterns: APL1 (GenBank Accession #JN180634) was highly expressed in fronds and APL3 (GenBank Accession #JN180636) showed the most abundance in developing turions. However, APL2 (GenBank Accession #JN180635) was not identified as DE gene due to only 1.5 times of difference at the time point of 0 and 3^rd^ day by the threshold value of 4 (Figure 3). We also compared the RNA-seq data of a fourth gene, tur4, with its developmental expression after ABA treatment [24]. The tur4 gene has the Gene ID Spipo7G0013500 in the sequenced genome of Spirodela. Although the tur4 gene responded to ABA treatment within hours, it appeared to return to nearly normal levels of expression thereafter. Northern blot analysis showed no induction at day 3 after ABA treatment, whereas we could still detect a 2-fold increase in tur4 expression with RNA-Seq, indicating that our method is more sensitive than Northern blot analysis. However, based on the developmental profile of the APLs and tur4 results, we found a cut-off for DE genes at 4-fold expressional changes the most meaningful.

### Response to ABA stimulus

The plant hormone abscisic acid (ABA) plays a major role as a signal in seed development and plant dormancy [42,43] and regulates many important aspects, such as the synthesis of seed storage proteins, starch and lipids [44,45]. In Spirodela, the exogenous ABA could easily trigger the dormant state (turions) from growth phase (fronds) [15]. We found 25 up-regulated DE genes belonging to gene families of “response to abscisic acid stimulus” and “negative regulation of abscisic acid mediated signaling pathway” (Table 3 and Additional file 1: Table S1). The pathway of ABA signal transduction and response seemed to be interwoven with enzyme metabolism (kinase, synthase, and phosphatase) and other signaling pathways (transporter, ethylene). Northern blot analysis shows that ABA rapidly up-regulates tur4 transcriptional level that encodes a peroxidase, which could stimulate turion formation and growth inhibition [24].

**Table 3:** FPKM for Up-regulated DE genes in response to ABA stimulus

**Gene ID**	**Fold change**	**Frond FPKM**	**Turion FPKM**	**Annotation**
Spipo6G0001100	146	0.3	45.3	Peripheral-type benzodiazepine receptor
Spipo5G0029200	57	0.8	48.1	Major facilitator superfamily protein
Spipo19G0014500	43	0.5	22.4	Galactinol synthase
Spipo26G0007700	17	8.4	140.0	Late embryogenesis abundant protein LEA
Spipo8G0058900	16	1.2	19.4	Flowering locus T/Terminal flower 1-like protein
Spipo4G0016300	15	15.2	235.2	Annexin
Spipo18G0029800	15	28.7	420.3	O-acetyltransferase-like
Spipo3G0078900	14	0.4	6.2	Stachyose synthase, putative
Spipo0G0155100	13	1.7	21.5	Ethylene-responsive transcription factor 1
Spipo0G0130700	9	1.6	15.5	C4-dicarboxylate transporter
Spipo7G0041900	7	1.4	9.8	ABC transporter G family member
Spipo3G0031800	7	10.3	74.2	Ethylene-responsive transcription factor 2
Spipo8G0062500	6	7.2	44.3	Receptor-like protein kinase
Spipo14G0026800	5	4.0	18.3	Eukaryotic aspartyl protease family protein
Spipo12G0003900	4	37.9	162.9	myb domain protein 73
Spipo5G0040500	8	23.1	189.7	Alpha-dioxygenase
Spipo0G0156500	6	15.5	97.9	Alpha-dioxygenase
Spipo0G0180000	6	98.0	561.3	Alpha-dioxygenase
Spipo0G0156600	5	19.5	104.4	Prostaglandin G/H synthase
Spipo8G0046200	67	0.2	15.6	Protein phosphatase 2c, putative
Spipo3G0013100	38	0.5	20.2	NAC domain-containing protein 67
Spipo23G0012800	32	1.1	34.2	Protein phosphatase 2C
Spipo21G0022300	10	6.1	59.1	Protein phosphatase 2c, putative
Spipo1G0021700	6	3.2	18.9	Protein phosphatase 2c, putative
Spipo6G0056800	4	11.5	46.5	NAC domain-containing protein 67

### Growth inhibition

Dormancy is generally defined by the lack of visible growth. The shoot apices cease active growth in perennial plants when a state of dormancy is reached. The seed dormancy is observed in seeds with a quiescent phase preventing germination. The same phenomenon was investigated for Spirodela in the presence and absence of growth. When we looked at DE genes associated with Spirodela growth by RNA-Seq data, we found genes of histone H3 (Spipo9G0039400, Spipo0G0046100 and Spipo13G0007500) and H4 (Spipo28G0019000), ribosomal protein (Spipo1G0126300), expansins (Spipo22G0026300), aquaporins (Spipo11G0033800, Spipo17G0045100), ribulose-1,5-bisphosphate carboxylase oxygenases (RuBisCO) (Spipo19G0027700, Spipo23G0013400) for carbon fixation were down-regulated in turions (Table 4). In eukaryotic cells, DNA replication requires the synthesis of histone proteins to package newly replicated DNA into nucleosomes. Expansins are a key endogenous regulator of plant cell enlargement [46]. Aquaporins support cell growth and especially contributes to cell expansion and cell division. The gene that is highly expressed in fronds (69 times higher than in turions) is aquaporin (Spipo11G0033800) (Table 4). Over-expression of aquaporin stimulates cell growth in tobacco [47] or in Arabidopsis [48]. These results further confirm our knowledge that fronds are mainly responsible for rapid growth through actively DNA assembly, protein synthesis and carbon fixation, leading to a quick biomass increase, in comparison to the turions, where these processes are greatly decreased. Previous studies also suggested this mechanism of the turion formation by measuring DNA, RNA and protein content, which showed that DNA, protein and RNA biosynthesis were largely inhibited, resulting in the decrease of cell division, expansion and differentiation [28].

**Table 4:** FPKM for Down-regulated DE genes associated with Spirodela growth

**Gene ID**	**Fold change**	**Frond FPKM**	**Turion FPKM**	**Annotation**
Spipo11G0033800	69	33.6	0.5	Aquaporin
Spipo17G0045100	5	86.3	17.8	Aquaporin
Spipo22G0026300	5	186.4	40.4	Expansin
Spipo9G0039400	7	68.2	9.4	Histone H3
Spipo0G0046100	7	112.4	16.4	Histone H3
Spipo13G0007500	6	159.5	27.5	Histone H3
Spipo28G0019000	5	77.9	14.8	Histone H4
Spipo3G0024800	14	1018.4	71.2	Pre-rRNA-processing protein PNO1
Spipo1G0126300	5	1371.4	293.1	60S ribosomal protein L10-like protein
Spipo19G0027700	29	6951.7	241.3	Ribulose bisphosphate carboxylase small chain
Spipo23G0013400	5	476.1	93.1	Ribulose-1 5-bisphosphate carboxylase/oxygenase activase

### Late embryogenesis abundant protein (LEA) genes are a valuable marker for dormancy

On the other hand, we found some specific mRNAs were increased in developing turions, for example LEAs. Although there were five members of LEA genes (Spipo14G0001200, Spipo5G0015500, Spipo0G0166800, Spipo1G0033500, Spipo26G0007700) with increased expression in turions, the LEA gene (Spipo0G0166800) was the most up-regulated DE gene; two other LEA genes (Spipo5G0015500 and Spipo14G0001200) were exclusively expressed in developing turions (Table 5). Indeed, the promoter of these LEA genes would be ideal to ensure expression of other coding regions exclusively in turions through transgenic approaches. Additionally, LEA was found to protect other proteins against desiccation, cold, and high salinity [49] and especially accumulates when plant seeds desiccate [50]. Given their high induction, they provide valuable markers for dormancy in general. In response to dehydration, endogenous ABA levels increased dramatically followed by induction of LEA [51]. As expected, when Spirodela fronds are destined to dormant turions triggered by ABA, desiccation is an indispensable step, in which LEA proteins play pivotal roles to preserve the cellular structures and nutrients in turions.

**Table 5:** FPKM for Turion-specific genes and DE transcriptional factors

** Gene ID**	**Fold change**	**Frond FPKM**	**Turion FPKM**	**Directionality**	**Annotation**
Spipo14G0001200	NA	0.0	31.0	Up-regulated	Late embryogenesis abundant protein LEA
Spipo5G0015500	NA	0.0	45.8	Up-regulated	Late embryogenesis abundant protein LEA
Spipo0G0166800	170	1.4	235.2	Up-regulated	Late embryogenesis abundant protein LEA
Spipo1G0033500	34	3.4	114.8	Up-regulated	Late embryogenesis abundant protein LEA
Spipo26G0007700	17	8.4	140.0	Up-regulated	Late embryogenesis abundant protein LEA
Spipo4G0008600	5	6.1	33.1	Up-regulated	bZIP transcription factor A
Spipo8G0037600	11	1.1	11.6	Up-regulated	Heat shock transcription factor A2
Spipo9G0002000	5	14.2	67.8	Up-regulated	Heat shock transcription factor A2
Spipo0G0155100	13	1.7	21.5	Up-regulated	Ethylene-responsive transcription factor 1
Spipo3G0031800	7	10.3	74.2	Up-regulated	Ethylene-responsive transcription factor 2
Spipo20G0027700	5	10.6	53.1	Up-regulated	Ethylene-responsive transcription factor 3
Spipo11G0028200	7	32.7	4.4	Down-regulated	Ethylene-responsive transcription factor 4
Spipo8G0045500	7	11.3	1.7	Down-regulated	WRKY transcription factor, putative
Spipo2G0055800	4	17.1	4.0	Down-regulated	bZIP transcription factor I

### Genes involved in carbon partitioning

Starch is the major carbon reserve in plant storage organs, and ABA has a signaling role by inducing starch biosynthetic gene expression and co-ordinate carbohydrate partitioning [52]. In our study, four genes (Spipo12G0062400, Spipo18G0038500, Spipo16G0027000 and Spipo27G0011300) (Additional file 1: Table S1) participating in starch biosynthesis were significantly enhanced in developing turions. The RNA-seq data was consistent with the qRT-PCR experiment of the key enzyme of large-subunit ADP-glucose pyrophosphorylase 3 (APL3) for starch biosynthesis that was highly expressed during turion development [13]. The RNA-Seq study for *Landoltia punctata* also revealed gene expression involved in starch biosynthesis was up-regulated under nutrient starvation [53]. Another way to accumulate starch content is to redirect carbon flow to starch biosynthesis. We found seven genes participate in the degradation of lipids by alpha- (Spipo0G0156600, Spipo0G0180000, Spipo0G0156500, Spipo5G0040500) or beta-oxidation (Spipo0G0179100, Spipo3G0031300, Spipo1G0110400), which probably allocate carbon to starch rather than fatty acids to achieve denser turions that sink to the bottom of streams during seasons (Additional file 1: Table S1). Previously, it has been shown that the carbon flow into seeds can be rebalanced between different macromolecules with different energy content [54]. Reallocation of carbon is critical for the improvement of oil production in novel crops in the future. In oilseed species, numerous biotechnological approaches have been carried out that were aimed to maximize the flow of carbon into oil by over-expression of enzymes of the TAG assembling network [55]. Although one might argue that turions would no longer be able to sink in water when filled with lipids, in those applications biomass would be accumulated under constant temperature.

Another way to investigate the balance of carbon partitioning can be derived from the average FPKM value (Fragments Per Kilobase of transcript per Million mapped reads) of all the key genes encoding both pathways. The genes encoding for lipid production were expressed relatively low with FPKM of 28 and 22 in fronds and turions, respectively. Therefore, the level of lipids remains low throughout development (Additional file 1: Table S2). Given the high level of starch in turions, genes in lipid production are not induced, whereas the ones for starch biosynthesis are during turion formation, providing us with a correlation between metabolic products and the regulation of the corresponding pathways. Given this correlation, we hypothesize that we could redirect carbon flow into lipids by blocking key genes of such as AGPS1, AGPL3, GBSSI and ACCase4, GPAT1, DGAT2, and over-express transcripts of the lipid pathway (Additional file 1: Table S2) together with turion-specific promoters, like LEAs (Spipo14G0001200, Spipo5G0015500, Spipo0G0166800) (Table 5).

### Turion-specific pathays

We found that the transcriptome also closely links the turion phenotypic variation with a thick cell wall and abundant secondary metabolites like pigment. The expressions of eight members of the UDP-glycosyltransferase superfamily (Spipo2G0010600, Spipo2G0043800, Spipo16G0044000, Spipo2G0039000, Spipo14G0034300, Spipo2G0124000, Spipo5G0014300, Spipo2G0077900) and two of the cellulose synthases (Spipo28G0017100, Spipo7G0044000) involved in cell call biosynthesis were increased (Additional file 1: Table S1). Three dihydroflavonol reductases (Spipo7G0010700, Spipo10G0000200, Spipo14G0054900) and one flavonoid 3’, 5’-hydroxylase (Spipo0G0155000) involved in the anthocyanin pathway were up-regulated (Additional file 1: Table S1). In addition, we found the average FPKM value for all key enzymes of lignin biosynthesis were 23 in fronds but 41 in turions, which may explain the rigidity of cell wall in turion cells to defend water pressure at the bottom of waters (Additional file 1: Table S2).

To gain a broad overview into the biological functions for DE genes, we next performed an analysis of gene ontology (GO) enrichment (Methods). We found a total of 24 enriched pathways (p < 0.01) in developing turions, whereas no enriched GO was found in fronds under the null hypothesis of the entire gene set of Spirodela (Young et al., 2010). The clustered DE genes were mainly related to response to ABA, fatty acid oxidation, and ion transportation. The GO functions of leaf senescence and cell wall modification were also highlighted (Table 6).

**Table 6:** Functional GO enrichment in developing turions

**Enriched GO ID**	**Description**
GO:0001561	Fatty acid alpha-oxidation
GO:0033539	Fatty acid beta-oxidation using acyl-CoA dehydrogenase
GO:0010167	Response to nitrate
GO:0015706	Nitrate transport
GO:0055114	Oxidation-reduction process
GO:0009830	Cell wall modification involved in abscission
GO:0009651	Response to salt stress
GO:0010106	Cellular response to iron ion starvation
GO:0010150	Leaf senescence
GO:0009737	Response to abscisic acid stimulus
GO:0006826	Iron ion transport
GO:0001676	Long-chain fatty acid metabolic process
GO:0001666	Response to hypoxia
GO:0046487	Glyoxylate metabolic process
GO:0071732	Cellular response to nitric oxide
GO:0010286	Heat acclimation
GO:0071446	Cellular response to salicylic acid stimulus
GO:0072329	Monocarboxylic acid catabolic process
GO:0019579	Aldaric acid catabolic process
GO:0009751	Response to salicylic acid stimulus
GO:0042542	Response to hydrogen peroxide
GO:0046686	Response to cadmium ion
GO:0009788	Negative regulation of abscisic acid mediated signaling pathway
GO:0009414	Response to water deprivation

### Transcriptional regulation of differentially expressed genes

Transcription factors (TFs) are crucial components of regulatory systems, which initiate vital changes in gene expression. Thus, we examined TF gene models and found nine TFs were significantly changed including two ABA-responsive element binding factors (bZIP, Spipo4G0008600 and Spipo2G0055800), four Ethylene-responsive element binding factors (ERFs, Spipo0G0155100, Spipo3G0031800, Spipo20G0027700 and Spipo11G0028200), two heat shock TFs (HSFs, Spipo8G0037600 and Spipo9G0002000), and one WRKY TF (Spipo8G0045500) (Table 5 and Additional file 1: Table S1).

### ABA-responsive element binding factor

**Figure 4:**
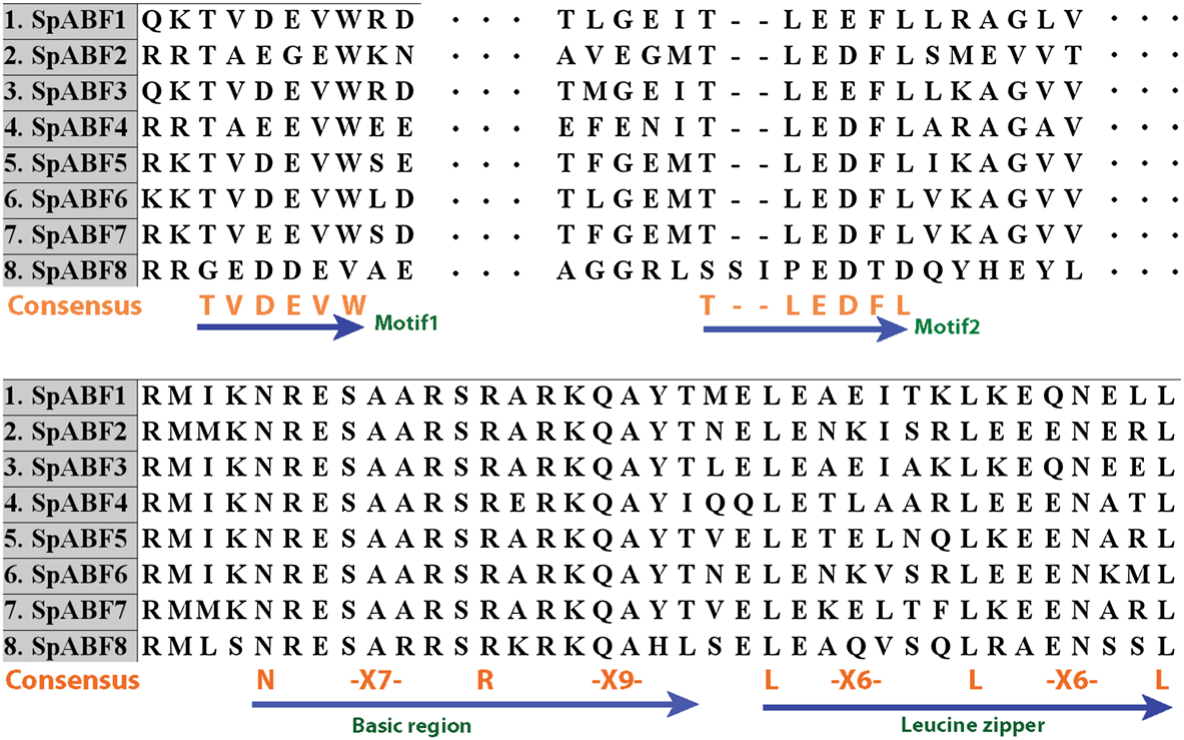
**Alignment of ABF domain from Spirodela.** The amino acid sequences of bZIP protein sequence from Spirodela were aligned and the conserved regions were demonstrated here. The consensus amino acids were labeled from conserved regions and highlight as motif 1 and motif 2, the primary structure of bZIP domains (basic region and leucine zipper). All members contain these four domains except SpbZIP, which only has a basic region and a leucine zipper. SpABF1-Spipo4G0008600; SpABF2-Spipo6G0055300; SpABF3-Spipo15G0021000; SpABF4-Spipo4G0111500; SpABF5-Spipo7G0034500; SpABF6-Spipo3G0017700; SpABF7-Spipo13G0002500; SpbZIP-Spipo2G0055800.

The bZIP trancription factors regulate plant development through a basic region and a leucine zipper dimerization motif that binds to DNA [56,57]. In the complete sequence of Spirodela genome [32], an exhaustive search of the bZIP superfamily was performed and 41 members identified. Among them, seven genes belong to the ABA-responsive element binding factors (ABFs), i.e., the bZIP superfamily group A due to their structural features with conserved regions C1-C2, basic regions, and leucine zippers (Figure 4) [56,58]. This group is thought to play a central role in controlling ABA-responsive gene expression in seeds and vegetative tissues via binding to ABA-responsive-elements (ABREs). For example, ABI5, one member of ABFs, induces LEA expression by binding to its promoters during seed maturation [58]. Here, all seven genes showed differentially increased expression levels, whereas only SpABF1 (Spipo4G0008600) was defined as a DE gene due to a significant change (Table 5). Noticeably, SpbZIP (Spipo2G0055800), another bZIP transcription factor, was significantly decreased in developing turions (Table 5). It shared leucine residues in the basic domain but missing other 2 conserved regions, corresponding to bZIP group I in Arabidopsis. Studies of group I genes from several species indicate that they might play a role in vascular development [56]. SpbZIP might positively regulate xylem and phloem development, too. Because both structure and function of turions are equivalent to seeds, less vascular tissue is needed in turions compared to fronds and the expression of SpbZIP is decreased accordingly. Thus, we conclude that a specific subset of bZIP transcription factors are involved in turion formation.

### Other TFs involved in ABA-mediated gene expression

**Figure 5:**
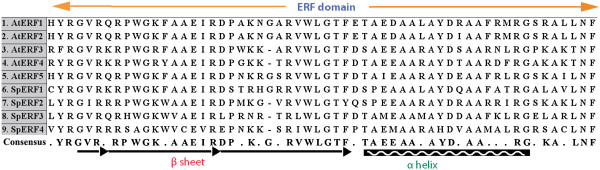
**Alignment of the ERF domain from Arabidopsis and Spirodela.** The bar and black arrows indicate β sheet motif, which interacts with the GCC box of target DNA. The cross-hatched box indicates the α helix. The consensus amino acids are underlined in ERF domain. The accession numbers are: AtERF1-BAA32418; AtERF2-BAA32419; AtERF3-BAA32420; AtERF4-BAA32421; AtERF5-BAA32422; SpERF1-Spipo0G0155100; SpERF2-Spipo3G0031800; SpERF3-Spipo20G0027700; SpERF4-Spipo11G0028200.

In addition to ABF TFs, other TFs were also identified to be involved in turion development. Ethylene-responsive element binding factors (ERFs) are transcription factors that are specific to plants. A highly conserved DNA binding domain, known as the ERF domain interacting directly with the GCC box in the ethylene-responsive-element (ERE), is the unique feature of this protein family [59] (Figure 5). ERFs also play a role in a variety of developmental processes such as flower, seed development [60], and fruit ripening [61]. We identified 57 ERF genes in the Spirodela genome, where SpERF1 (Spipo0G0155100), SpERF2 (Spipo3G0031800), and SpERF3 (Spipo20G0027700) were significantly up-regulated and SpERF4 (Spipo11G0028200) down-regulated in response to turion development (Table 5). It had been reported that AtERF1, AtERF2, ATERF5 functioned as activators of GCC box-dependent transcription in Arabidopsis leaves, but AtERF3 and AtERF4 acted as repressors [57,59]. It also was shown that ERF2 and ERF4 enhanced the transcription of a reporter gene in tobacco protoplasts [62]. The three highly up-regulated ERFs in Spirodela turions should therefore play an important role in turion development.

Heat shock transcription factors (HSFs) are transcriptional activators of heat shock genes. An increasing number of studies indicated that some HSFs appeared during the maturation stage of the seed, when cell division ceased and seeds adapted to desiccation and long-term survival [63]. Here, the increased expression of two HSFs (Spipo9G0002000 and Spipo8G0037600) (Table 5) might also indicate an important function for turion desiccation and survival during long periods of winter.

WRKY transcription factors (TFs) are key regulators of many plant processes, including the responses to biotic and abiotic stresses, senescence, seed dormancy, and seed germination [64]. *In vivo* and *in vitro* promoter-binding studies showed that WRKY TFs could either activate or repress the expression of downstream ABFs through W-box sequences present in their promoters [65]. However, whether the Spirodela WRKY TF (Spipo8G0045500) (Table 5) is a repressor or activator needs to be further investigated.

Together, the significant changes in the expressions of ABFs, ERFs, HSF and WRKY TF reflected their obligatory regulation during turion development. Their involvement in the transition from fronds to turions and their control of spatial and temporal expression of target genes provides us also with new tools to create specialized traits through tailoring of chimeric genes.

### *cis-*element

Control of gene expression is achieved through the binding of transcription factors to specific *cis-*elements in promoter regions of target genes [66]. To predict potential pairs of TFs and *cis*-elements, we scanned a 1-kb region upstream of DE genes with the PLACE database [27]. We found 30 up-regulated DE genes containing the *cis-*element of ABA-responsive element (ABRE: YACGTGGC) and 119 with ethylene-responsive element (ERE: GCCGCC) (Additional file 1: Table S1). These target genes of ABFs and ERFs are associated with seed dehydration (like late embryogenesis abundant proteins), regulatory transcription factor, protein kinases and phosphatases (like CPK, MAPK), carbohydrate and secondary metabolism (like cellulose synthase and stachyose synthase), and senescence-associated proteins (like Glutathione-S-transferase).

## Discussion

**Figure 6:**
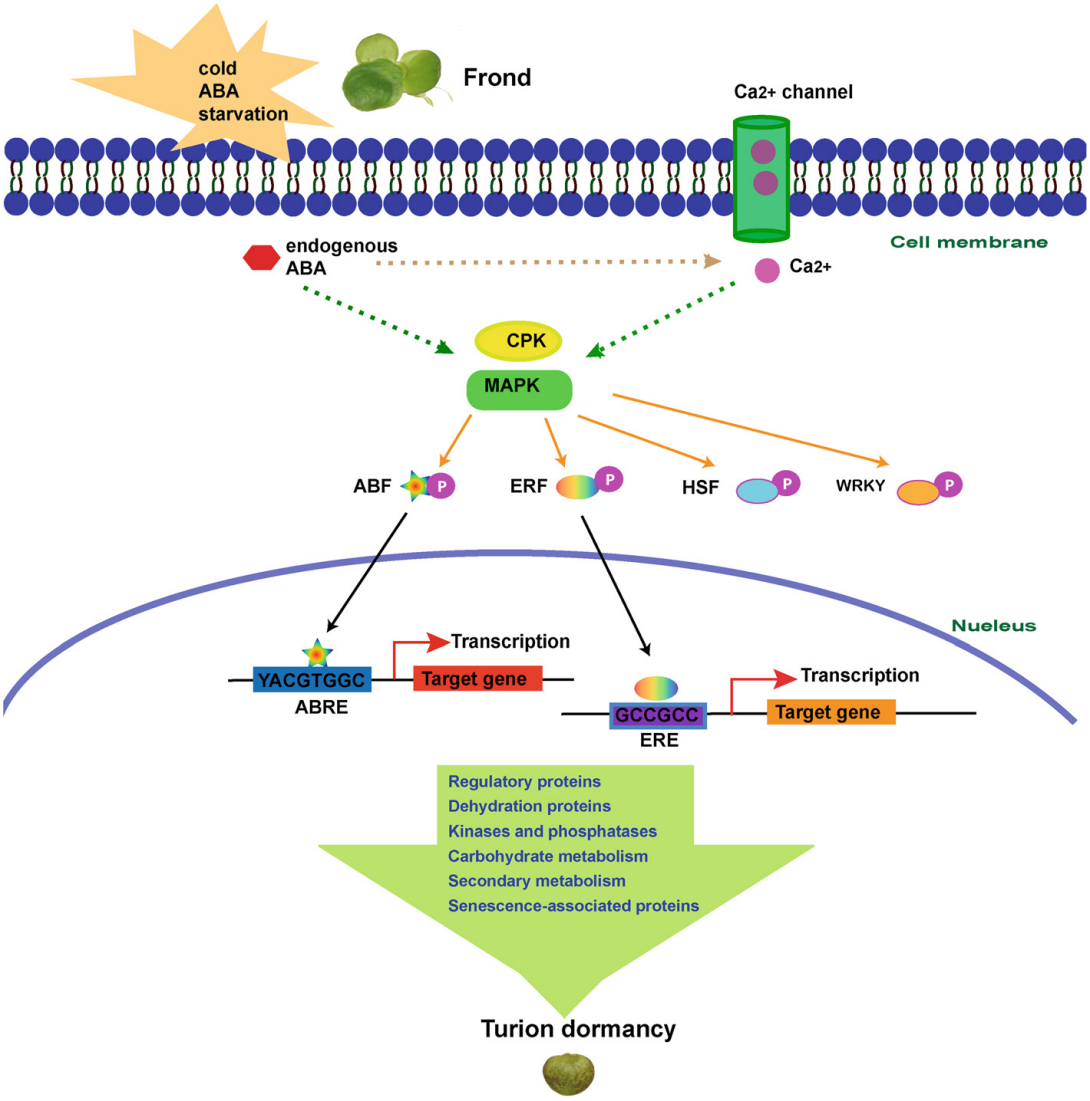
**A model of development of Spirodela dormancy through the signal transduction in response to environmental stimuli.** Phosphorylated proteins are labeled as pink circles with a P inside. Solid lines represent direct connections. The dotted line indicates indirect connection. Not all linkages and details of pathway are shown in this diagram in order to simplify the model. Abbreviations: ABA (abscisic acid), CPK (calcium-dependent protein kinase), MAPK (mitogen-activated protein kinase), ABF (ABA-responsive element binding factor), ERF (ethylene-responsive element binding factor), HSF (heat shock transcription factor), WRKY (WRKY transcription factors), ABRE (ABA-responsive element), ERE (ethylene-responsive element).

ABA is essential for seed maturation and also enforces a period of seed dormancy so that the seeds do not germinate prematurely during unseasonably conditions. The same behavior is seen in dormant Spirodela turions that are induced by low temperature, limited nutrition, or exogenous ABA. The external stimuli rapidly induce both Ca^2+^ influx and endogenous ABA synthesis [67]. In maturing seed, ABA-regulated genes include those required for the synthesis of storage reserves and the acquisition of desiccation tolerance. Ca^2+^ can act as secondary messenger to activate the expression of cascade components of calcium-dependent protein kinase (CPK) and mitogen-activated protein kinase (MAPK). The structure of CPK shows there are four Ca^2+^-binding EF hand domains allowing the protein to function as a Ca^2+^ sensor. In addition to Ca^2+^, reversible phosphorylation also regulates kinase activity [68]. A number of studies have demonstrated that MAPKs in Arabidopsis are associated with hormone biosynthesis and signaling including ethylene and ABA [43]. Both of CPK and MAPK could phosphorylate a wide range of target proteins, including other kinases and/or transcription factors [44,57], in particular SpERF of Spipo0G0155100, Spipo3G0031800 and Spipo20G0027700, SpABF of Spipo4G0008600 and Spipo2G0055800, SpHSF of Spipo8G0037600 and Spipo9G0002000, and SpWRKY of Spipo8G0045500 (Table 5). The activation of TFs ultimately regulates their target genes to cease cell division but begin to accumulate secondary metabolites. As shown in flowering seeds, aspects of reserve accumulation and late embryogenesis abundant (LEA) gene expression are controlled largely by the coordinated action of transcription factors [44]. Taken together, we generated a model summarizing the signal transduction leading to Spirodela dormancy based on integration of our results and previous knowledge (Figure 6).

## Conclusions

Many studies have been concerned with seed development in plants. Seeds are the product of sexual reproduction and the segregation of Mendelian traits. They also represent a dormant state in the life cycle of the plant and they compartmentalize nutrients for growth in the absence of photosynthesis. Agriculture could not exist without these properties of plants. Here, we studied a plant that propagates by clonal division and can undergo dormancy without forming seeds. The aquatic plant Spirodela could not survive on water surface without human intervention, when the water freezes. It simply switches to dormancy and accumulates starch that allows it to sink to the bottom of the water to escape the ice. Besides low temperature, however, the same switch can be achieved with the hormone ABA that has been shown to perform the same change for seed maturation. Using such an induction with Spirodela, we can study genes that regulate dormancy. Here, we isolated total RNA, excluded ribosomal RNA before and at the onset of dormancy, sequenced them with next-generation technology, and identified the transcripts by mapping them back to the genome sequence. The detailed analysis of the transcriptional landscape of differentially expressed genes provides the first comprehensive view at the dormancy of aquatic plants. On the other hand, research studies have been initiated with the goal of developing duckweed species as an alternative to algae for oil production with the fact of fast growth and quick biomass accumulation [69]. The expression data for lipid and starch biosynthesis together with the turion-specific transcriptional genes from our RNA-Seq data would be the ideal targets to develop duckweeds into oil crops.

## Abbreviations

ABA: Abscisic acid; FPKM: Fragments per kilobase of transcript per million mapped reads; DE gene: Differentially expressed genes; LEA: Late embryogenesis abundant protein; ABF: ABA-responsive element binding factors; ERF: Ethylene-responsive element binding factors; CPK: Calcium-dependent protein kinase; MAPK: mitogen-activated protein kinase; CCR: Cinnamoyl-CoA reductase; CAD: Cinnamyl-alcohol dehydrogenase; APS: ADP-glucose pyrophosphorylase small subunit; APL: ADP-glucose pyrophosphorylase large subunit; SS: Starch synthase; GBSS: Granule-bound starch synthase; BE: Starch branching enzyme; DBE: Starch debranching enzyme; ACCase: Acetyl-CoA carboxylase; GPAT: Glycerol-3-phosphate acyltransferase; AGPAT: Acylglycerophosphate acyltransferase; DGAT: Diacylglycerol acyltransferase.

## Competing interests

The authors declare that they have no competing interests.

## Authors’ contributions

Conceived and designed the experiments: WW Performed the experiments: YW Analyzed the data: WW Wrote the paper: WW YW JM Supervised the work: JM. All authors read and approved the final manuscript.

## Supplementary Material

Additional file 1: Table S1 Annotation for 362 up- or down-regulated DE genes with FPKM values and fold changes. The genes discussed in main text were labeled as bold. The genes with *cis*-element of ABRE or ERE were indicated with “YES”. GO annotations involved in ABA response or starch biosynthesis or fatty acid oxidation were also marked accordingly. **Table S2**: Expression patterns indicated by FPKM value of key genes for lignin, starch and lipid biosynthesis. Click here for file
